# Graphene-Wrapped Anatase TiO_2_ Nanofibers as High-Rate and Long-Cycle-Life Anode Material for Sodium Ion Batteries

**DOI:** 10.1038/srep13862

**Published:** 2015-09-10

**Authors:** Yeolmae Yeo, Ji-Won Jung, Kyusung Park, Il-Doo Kim

**Affiliations:** 1 Department of Materials Science and Engineering, Korea Advanced Institute of Science and Technology (KAIST), 291 Daehak-ro, Yuseong-gu, Daejeon 305-701, Republic of Korea; 2 Texas Materials Institute, The University of Texas at Austin, Austin, Texas 78712, United States

## Abstract

Anatase TiO_2_ has been suggested as a potential sodium anode material, but the low electrical conductivity of TiO_2_ often limits the rate capability, resulting in poor electrochemical properties. To address this limitation, we propose graphene-wrapped anatase TiO_2_ nanofibers (rGO@TiO_2_ NFs) through an effective wrapping of reduced graphene oxide (rGO) sheets on electrospun TiO_2_ NFs. To provide strong electrostatic interaction between the graphene oxide (GO) sheets and the TiO_2_ NFs, poly(allylamine hydrochloride) (PAH) was used to induce a positively charged TiO_2_ surface by the immobilization of the -NH_3_^+^ group and to promote bonding with the negatively charged carboxylic acid (-COO^−^) and hydroxyl (-O^−^) groups on the GO. A sodium anode electrode using rGO@TiO_2_ NFs exhibited a significantly improved initial capacity of 217 mAh g^−1^, high capacity retention (85% after 200 cycles at 0.2C), and a high average Coulombic efficiency (99.7% from the second cycle to the 200^th^ cycle), even at a 5C rate, compared to those of pristine TiO_2_ NFs. The improved electrochemical performances stem from highly conductive properties of the reduced GO which is effectively anchored to the TiO_2_ NFs.

Lithium-ion batteries (LIBs) have been widely used as power sources for portable electronic devices and hybrid electric vehicles. However, the growing usage and application of LIBs bring a fundamental question as to whether lithium resources on earth can cover future industrial demands. For this reason, many researchers have studied other energy-storage devices beyond LIBs. The sodium-ion batteries (NIBs) are a promising candidate because it is similar to the conventional LIB system in terms of the charge/discharge mechanism and the cell configuration. A number of notable studies of NIB have been conducted recently in search of cost-effective, high-capacity, and structurally/electrochemically stable sodium-ion electrode materials^1^,^2^,^3^,^4^.

Recently, tin (Sn)^5^,^6^, antimony (Sb)^7^ and red phosphorus (red P)^8^,^9^ were reported as promising sodium-ion anode materials^10^. They have high theoretical specific capacities of 847 mAh g^−1^ for Na_15_Sn_4_^11^, 660 mAh g^−1^ for Na_3_Sb^11^ and 2596 mAh g^−1^ for Na_3_P^8^. However, these high-capacity anode materials undergo large volume changes during cycling, which can generate pulverization or cracks, eventually leading to cell failure. For example, after sodiation, the theoretical volumetric expansions of Na_15_Sn_4_^6^, Na_3_Sb^11^, and Na_3_P^8^ were approximately 420%, 390% and 308%, respectively. As experienced in the LIB system, the volumetric expansion problem is difficult to solve because it stems from the intrinsic properties of the materials, which are related to structural changes during sodiation. Consequently, alternative anode materials which undergo minimal volume changes should be found for the practical application of NIBs.

In this regard, TiO_2_ is a particularly interesting anode material. Xu *et al.* first reported anatase TiO_2_ (hereafter, TiO_2_) for a sodium-ion battery with a stable cycle life of 100 cycles^12^. After this study, TiO_2_ received much attention as a promising sodium-ion anode material^13^. In-depth studies of the electrochemical reaction mechanism between the Na^+^ and TiO_2_ have also been conducted^14^,^15^. It is believed that TiO_2_ stores Na^+^ below 0.8 V through the Ti^4+^/^3+^ redox reaction, which is based on Na^+^ insertion in the host structure. Then, the metastable sodium titanate phase is converted into metallic titanium, sodium superoxide and an amorphous sodium titanate phase at 0.3 V vs Na/Na^+^ during cycling. One major concern about TiO_2_ is its low electrical conductivity owing to its high bandgap of ~3.2 eV, which gives rise to the insulating nature of intrinsic TiO_2_ without a dopant^16^. In order to improve the electrical transport characteristics of TiO_2_, several studies have been performed to achieve nanostructural TiO_2_ (i.e., nanoparticles, nanorod, nanotube)^17^,^18^,^19^ with advanced carbonaceous materials such as carbon nanotubes (CNTs)^20^ or graphene^21^. A carbon-modified TiO_2_ composite showed a noticeable improvement in its electrochemical performance, but it still had problems such as a high cost and low productivity due to its complex manufacturing process.

In particular, for LIB applications, graphene-TiO_2_ composite structures, including those with TiO_2_ particles decorated onto the surface of graphene^22^,^23^, stacked TiO_2_ and graphene layers^24^, structures with physically mixed TiO_2_ particles and graphene^25^, and those with TiO_2_ particles wrapped with graphene^26^ have been widely studied. However, a simple mixing route between carbon/graphene and zero-dimensional (0D) oxide nanoparticles often requires large amounts of carbon/graphene. Severe aggregation of the oxide nanoparticles or the graphene itself is easily observed during the mixing process. On the other hand, well-interconnected one-dimensional (1D) nanostructures can greatly improve the electrochemical kinetics owing to a reduced diffusion length to the fiber core (t = L^2^/D; t: reaction time, L: ion diffusion length, D: diffusion coefficient)^27^. For such a 1D nanostructure, intriguingly, the graphene-wrapping route offers significantly improved cycle performance and rate capability with a small amount of graphene and without the aggregation of the graphene sheets. In this study, we propose graphene-wrapped 1D TiO_2_ nanofibers (hereafter, TiO_2_ NFs) for the first time as a high-rate and long-cycling anode material for sodium-ion batteries. In this study, 1D TiO_2_ NFs were prepared via an electrospinning method, and poly (allylamine hydrochloride) (PAH) was used as a surface modifier to induce a positively charged TiO_2_ surface, i.e., -NH_3_^+^-grafted TiO_2_ NFs^28^. Then, the graphene-wrapping process was done to obtain graphene-wrapped TiO_2_ NFs. The electrochemical sodiation/desodiation properties of the graphene-wrapped TiO_2_ NFs and their reaction mechanism are discussed.

## Results

### Schematic illustration of the electrospinning and graphene-wrapping process

**Figure 1:**
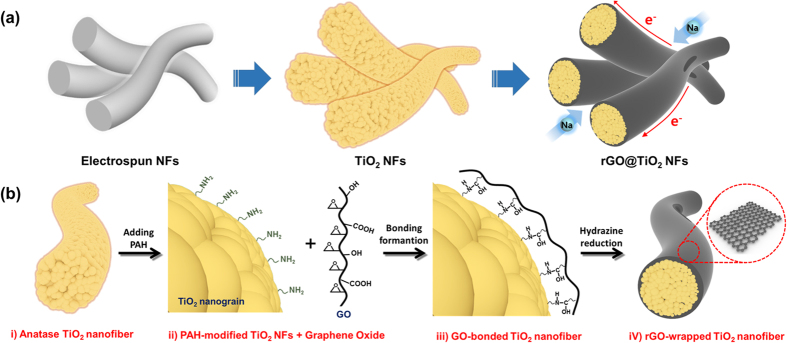
Synthesis of graphene-TiO_2_ NFs. (**a**) products at each synthetic step: as-spun NFs by electrospinning, anatase TiO_2_ NFs after calcination at 500 ^o^C for 1 h, and rGO@TiO_2_ NFs by graphene-wrapping. (**b**) graphene-wrapping mechanism: i) the surfaces of the as-prepared anatase TiO_2_ NFs are functionalized to amine groups with an aqueous PAH solution (PAH-modified TiO_2_ NFs), ii) the GO-TiO_2_ NF composite solution was formulated by adding a GO solution to a PAH-TiO_2_ NF solution, iii) Strong bonding formation between GO and TiO_2_ NFs through cross-linking, and iv) GO reduced by hydrazine to obtain the rGO@TiO_2_ NF solution. Proper centrifugation and drying followed after each step. This figure was drawn by one of co-authors.

Figure 1 shows the processing steps for the synthesis of the reduced graphene-oxide-wrapped TiO_2_ NFs (hereafter, rGO@TiO_2_ NFs). The rGO@TiO_2_ NFs were obtained by several synthetic steps, and the products in each step are shown in Fig. 1a. First, as-spun Ti precursor/polymer composite NFs were obtained via an electrospinning method. After high-temperature calcination, the TiO_2_ NFs were formed by the thermal decomposition of the matrix polymer and the crystallization of the TiO_2_ particles composing the NFs. With regard to the graphene-wrapping method, its mechanism is illustrated in Fig. 1b. In order to provide strong electrostatic interaction between the negatively charged graphene oxide (GO) and the as-prepared TiO_2_ NFs above, (i) we grafted the surfaces of the TiO_2_ NFs by using poly (allylamine hydrochloride) (PAH). TiO_2_ NFs were positively charged by -NH_3_^+^ in the solution; (ii) Subsequently, GO sheets were added to the TiO_2_ NF-dispersed solution, and the solution was mechanically agitated to ensure homogeneous mixing. GO sheets have sufficient functional groups such as carboxylic acid (-COOH) and hydroxyl (-OH) groups, which induce surface-negative charges (-COO^−^ and -O^−^) in the solution. Then, the positively charged TiO_2_ NFs and the negatively charged GO are self-assembled; (iii) Crosslinking between GO and PAH arises due to ring-opening of the epoxy groups of GO as well as partial contribution of the carboxylic group, originated from the nucleophile reaction of the unpaired electrons of the amine groups^28^; (iv) Finally, hydrazine was added to the mixed solution including the GO and PAH-modified TiO_2_ NFs to transform the GO sheets into reduced GO (rGO) sheets. This graphene-wrapping mechanism was discussed in our previous report^29^. As part of the processes above, proper centrifuging and drying were conducted.

### Characteristics of TiO_2_ NFs and rGO@TiO_2_ NFs

**Figure 2:**
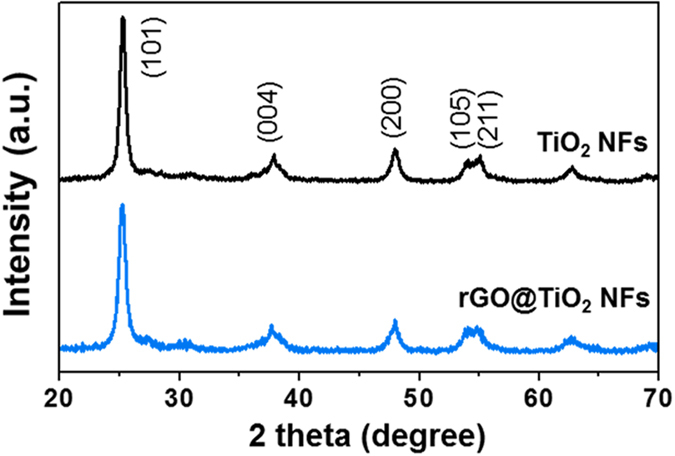
X-ray diffraction patterns of TiO_2_ NFs and rGO@TiO_2_ NFs.

Figure 2 shows the X-ray diffraction (XRD) patterns of the TiO_2_ NFs and the rGO@TiO_2_ NFs. The XRD patterns of both samples confirm that they have the original anatase TiO_2_ structure (space group I4_1_/amd, JCPDS PDF#21–1272). The main peak of the anatase TiO_2_ at 25.2^o^ indicates the crystal lattice plane (101), and other strong peaks at 37.8^o^, 48^o^, 54.8^o^, and 55^o^ present the (004), (200), (105) and (211) planes, respectively. Moreover, it is important to note that the graphene-wrapping process did not affect the crystal structure of the TiO_2_. Using the Scherrer equation, 
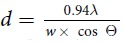
 we estimated the mean crystallite size of the nanoparticles comprising the polycrystalline TiO_2_ NFs. In equation 1, *d* is the mean grain size, λ is the wavelength of the Cu K_α_ radiation (0.154 nm), *θ* is the Bragg angle considered, and *w* is the line width at half-maximum intensity on the 2 *θ* scale, in radians. From this calculation, we estimated the average crystallite size of the anatase TiO_2_ to be 15.5 nm.

**Figure 3:**
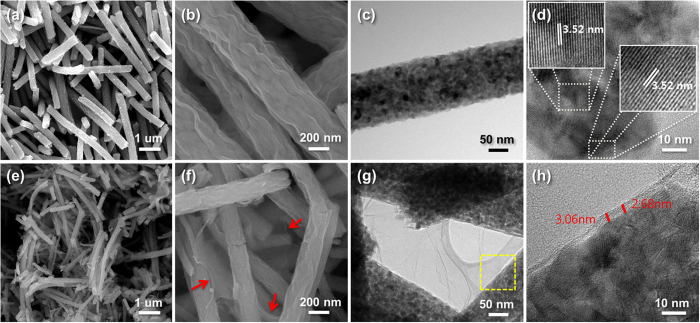
(**a**–**b**) SEM and (**c**–**d**) TEM images of TiO_2_ NFs; (**e**–**f**) SEM and (**g**–**h**) TEM images of rGO@TiO_2_ NFs. (**h**) shows an HRTEM image of the yellow frame in (**g**). Lattice of the anatase TiO_2_ (inset images); the graphene connection (red arrows) and thickness of the graphene wrapping (red lines) are well shown.

The morphological features of the TiO_2_ NFs and rGO@TiO_2_ NFs were characterized by scanning electron microscopy (SEM) and transmission electron microscopy (TEM), respectively. As shown in the SEM images in Fig. 3a,b, calcined TiO_2_ NFs with a diameter of approximately 200–300 nm exhibited a wrinkled surface and a straight line shape. The high-resolution TEM (HRTEM) image of the calcined TiO_2_ NFs clearly confirms that the Ti precursor was crystallized into polycrystalline TiO_2_, which is composed of small nanoparticles (Fig. 3c,d). The lattice fringes of the TiO_2_ (3.52 Å) NF correspond to the TiO_2_ (101) plane (JCPDS PDF#21–1272). Figure 3e,f depict SEM images of the rGO@TiO_2_ NFs. The rGO sheets cover all the surfaces of the TiO_2_ NFs well and effectively interconnect the TiO_2_ NFs to each other. Figure 3g exhibits a TEM image of the edge structure of the rGO@TiO_2_ NFs. We note the HRTEM image in Fig. 3h, which is a highly magnified image of the yellow frame in Fig. 3g and which reveals that the thickness of the rGO sheets is approximately 3 nm.

**Figure 4:**
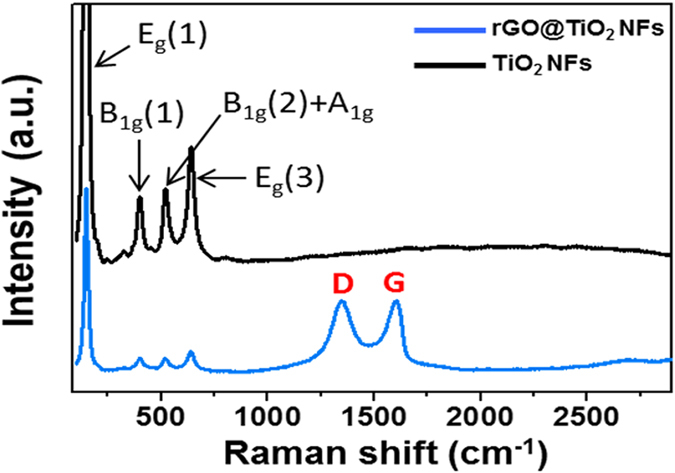
Raman spectra of TiO_2_ NFs and rGO@TiO_2_ NFs.

To provide further investigation, we conducted Raman and FT-IR analyses of both the TiO_2_ NFs and rGO@TiO_2_ NFs. The Raman spectra of the TiO_2_ NFs exhibit peaks which are located at approximately 145, 398, 520 and 640 cm^−1^ (Fig. 4). These peaks correspond to the lattice vibrational model of the E_g_(1), B_1g_(1), B_1g_(2) + A_1g_ and E_g_(3) bands of the anatase TiO_2_, respectively^30^. The Raman spectra of the rGO@TiO_2_ NFs equally contain the E_g_(1), B_1g_(1), B_1g_(2) + A_1g_ and E_g_(3) bands of the anatase TiO_2_, which also confirms that the graphene-wrapping process does not cause any local structure changes. Likewise, the rGO@TiO_2_ NFs show two peaks of rGO at 1349 and 1605 cm^−1^. These two peaks denote the general G and D bands of rGO, respectively^31^. The Raman analysis clearly verified that the structural properties of the TiO_2_ NFs, which were well covered by rGO, did not change after the graphene-wrapping process.

**Figure 5:**
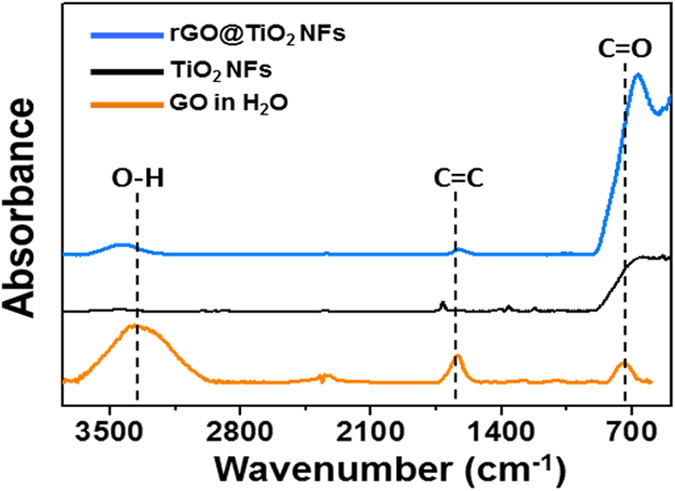
FT-IR spectra of TiO_2_ NFs, rGO@TiO_2_ NFs and the GO solution in H_2_O.

Figure 5 presents the Fourier-transformed infrared (FT-IR) spectra of the TiO_2_ NFs, the rGO@TiO_2_ NFs, and GO in H_2_O. The GO in H_2_O was measured in the attenuated total reflection (ATR) mode. Both the TiO_2_ NFs and rGO@TiO_2_ NFs showed Ti-O and Ti-O-Ti vibrations at 500–700 cm^−1^ 32, which provides evidence of the structural identity of these two materials. Furthermore, several bands located at a similar wavenumber in both the rGO@TiO_2_ NFs and the GO in H_2_O revealed that the graphene-wrapping process was successful. The broad band on the rGO@TiO_2_ NFs overlaps with the C = O stretch of the carbonyl groups at the edges of GO at 740 cm^−1^. Moreover, the C = C stretch at 1630 cm^−1^ and the bending vibration O-H stretch at 3100–3500 cm^−1^ are apparent in both the rGO@TiO_2_ NFs and the GO in H_2_O^31^.

### Electrochemical reaction with Na^+^

**Figure 6:**
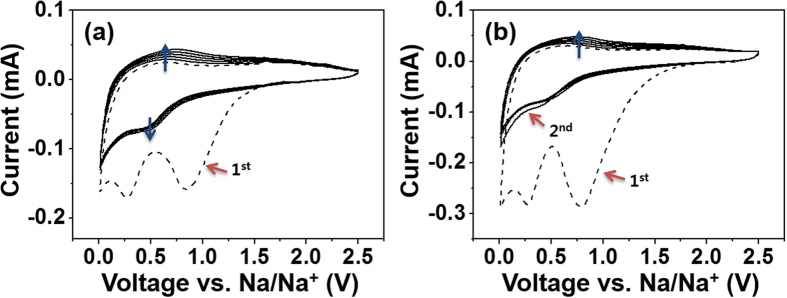
Cyclic voltammetry of (a) TiO_2_ NFs and (b) rGO@TiO_2_ NFs tested at a scan rate of 0.5 mV s^−1^ for six cycles.

Figure 6a,b show the cyclic voltammetry (CV) curves of the TiO_2_ NFs and the rGO@TiO_2_ NFs. A CV test was performed at a scan rate of 0.5 mV s^−1^ at 0.01 ~ 2.5 V for six cycles.

In the first cathodic scan of the TiO_2_ NFs and the rGO@TiO_2_ NFs, first irreversible reduction peaks at approximately 0.84 V are assigned to the formation of a solid electrolyte interface (SEI), and the second reduction peaks and the third peaks near 0.26 V and 0.02 V are attributed to sodiation and electrolyte decomposition. With subsequent cyclic sweeps, for both the TiO_2_ NFs and the rGO@TiO_2_ NFs, the second cathodic peaks and anodic peaks shift to higher potentials (~0.5 V and ~ 0.75 V). These phenomena may be caused by redox couple of Ti^4+^/Ti^3+^. Such results were already observed in previous study reported by Wu *et al.*^33^ Upon further cathodic and anodic voltage sweeps, broad cathodic and anodic peaks near 0.5 V and 0.75 V were reversely observed in both electrodes. This result reveals that both anode materials present the same electrochemical characteristics, especially in terms of reversibility.

## Discussion

### *Ex-situ* analysis of a plausible reaction mechanism

**Figure 7:**
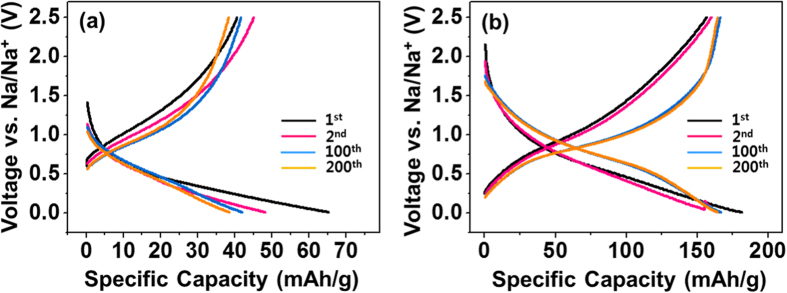
Charge and discharge curves of (a) TiO_2_ NFs and (b) rGO@TiO_2_ NFs at a 1 C (335 mA g^−1^) rate.

The charge-discharge voltage curves of the electrodes using TiO_2_ NFs and rGO@TiO_2_ NFs at a 1C rate (335 mAh g^−1^) were measured after one cycle at 10 mA g^-1^ (Supplementary Figures S1a,b). During the first cycle at 10 mA g^−1^, the voltage curves of the two electrodes showed two plateau regions near 1.25 V and 0.2 V while discharging. These two plateau regions are ascribed to the formation of a SEI layer, which is in good agreement with the results of the initial CV curves in Fig. 6. In the 1^st^, 2^nd^, 100^th^, and 200^th^ cycle during the following 1C rate charge/discharge, most of the capacities of the TiO_2_ NFs and rGO@TiO_2_ NFs are below 0.6 V vs. Na/Na^+^ (Fig. 7a,b). The rGO@TiO_2_ NFs showed enhanced capacity levels at all cycles and greatly improved capacity retention as compared to the TiO_2_ NFs after 200 cycles; these results were 90% and 58% in the rGO@TiO_2_ NFs and TiO_2_ NFs, respectively. From the voltage profiles, we found that no voltage plateau appears in any of the cycles, which differs from the reported voltage profile of anatase TiO_2_ for LIBs^34^. In typical anatase TiO_2_, the redox potential for Li^+^ insertion/deinsertion is about 1.6 V versus Li/Li^+^; this can be written as follows:

TiO_2_ + xLi^+^ + xe^−^ ↔ Li_x_TiO_2_

In this regard, we studied the charge/discharge behaviors using TiO_2_ NFs and rGO@TiO_2_ NFs as anodes for LIBs to determine whether the smooth voltage profiles are caused by our choice of materials. The experiment using lithium metal as a counter electrode was performed at 1C rate after pre-cycling at 10 mA g^−1^ (Supplementary Figure S2). In this experiment, both the TiO_2_ NFs and rGO@TiO_2_ NFs exhibited a Li^+^ ion insertion plateau near 1.6 V, as reported previously^35^. This finding verifies that the reaction mechanism of TiO_2_ NFs and rGO@TiO_2_ NFs for lithium insertion is based on insertion, as predicted. However, in the sodium insertion case, the TiO_2_ NFs and rGO@TiO_2_ NFs indicate smooth charge/discharge voltage behaviors, which is fundamentally different from the case of lithium insertion. Although the effect of the particle size could cause some difference in the plateau length, generally the size effect does not eliminate this plateau. Therefore, it is important to note that the reaction mechanism of TiO_2_ for NIBs may differ from that of LIBs.

**Figure 8:**
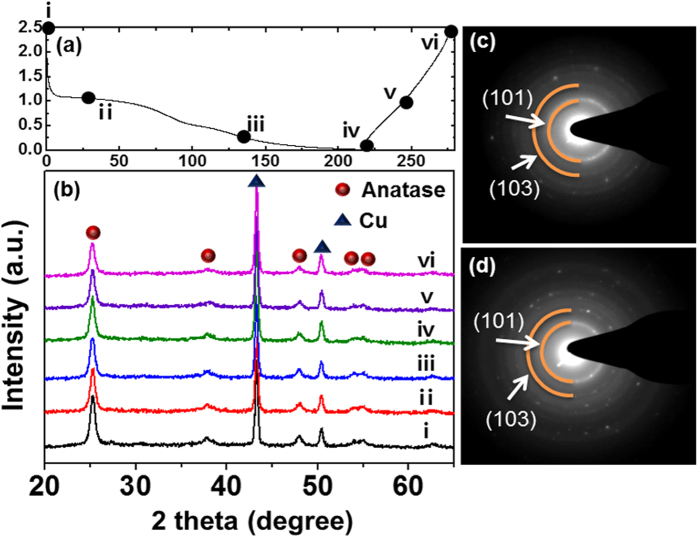
(**a**) First cycle voltage profile (X axis-specific capacity/Y axis-potential); (**b**) *Ex-situ* XRD data (Discharge; pristine, 1 V, 0.1 V, fully discharged. Charge; 1 V, fully charged) and SAED pattern with a TEM images in (**c**) fully discharged and (**d**) fully charged states.

To confirm the plausible reaction mechanism of anatase TiO_2_ for sodium insertion/extraction, we performed *ex-situ* XRD and *ex-situ* TEM analyses. Figure 8a presents the first cycle voltage profile at a 0.2C rate. Each Roman numeral indicates the cut-off voltage for the *ex-situ* analyses. Figure 8b shows *ex-situ* XRD patterns of pristine TiO_2_ NFs at 1 V, 0.1 V and 0.01 V while discharging and at 0.1 V and 2.5 V while charging. We could not find any significant structural decomposition through the *ex-situ* XRD analysis, and thus this may be interpreted as that the anatase structure was maintained during cycling. An *ex-situ* TEM analysis was performed to determine the structural stability. Figure 8c,d indicate selected-area electron diffraction (SAED) patterns in fully discharged and fully charged states, respectively. Our results rather coincide with a recent report by Kim *et al.*^36^ Kim reported that the anatase structure is maintained during extensive cycling and suggested that the reaction mechanism is insertion through an X-ray absorption spectroscopy (XAS) analysis. This may differ from the results reported by Wu *et al.*, who claimed that the XRD reflections of the anatase phase disappear in the discharged state. Although we observed that the (004) and (200) reflections at 38^o^ and 48^o^ still exist after a full discharge in our case, the results in here remain controversial. Therefore, a further elaborate analysis is necessary for a precise investigation of the reaction mechanism of graphene/TiO_2_ hybrid materials.

### Electrochemical performances

**Figure 9:**
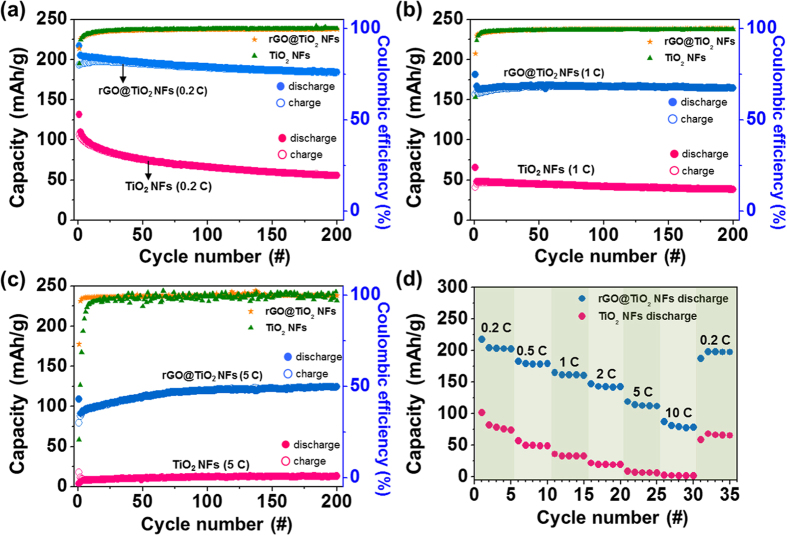
Charge–discharge capacity and coulombic efficiency vs. the cycle number for TiO_2_ NFs and rGO@TiO_2_ NFs tested (**a**) at a rate of 0.2C (67 mA g^−1^), (**b**) at a rate of 1C (335 mA g^−1^) and (**c**) at a rate of 5C (1675 mA g^−1^). (**d**) Rate capabilities of TiO_2_ NFs and rGO@TiO_2_ NFs evaluated at various rates of 0.2C, 0.5C, 1C, 2C, 5C and 10C.

The galvanostatic cycles of Na-anode electrodes using TiO_2_ NFs and rGO@TiO_2_ NFs were tested at rates of 0.2C (67 mA g^−1^), 1C (335 mA g^−1^) and 5C (1.675 A g^−1^) for 200 cycles (Fig. 9a–c). All cycle tests were performed after an initial charge/discharge process at 10 mA g^−1^. During the galvanostatic cycles, the rGO@TiO_2_ NFs maintained significantly higher specific capacities than those of the TiO_2_ NFs. For example, at the rates of 0.2C, 1C, and 5C, the rGO@TiO_2_ NFs exhibits initial discharge capacities of 217, 165 and 124 mAh g^−1^, respectively. Previously, Cha *et al.* showed through cyclic voltammetry that sodium ions could be stored in graphene^21^. In other words, graphene could serve as an electrochemically active material. However, in this study, we found through an elemental analysis (EA) that the detected carbon element in rGO@TiO_2_ NFs was only 1.85 wt% (Supporting Information, Table S1). This negligible amount of carbon coming from graphene sheets may not appreciably contribute to the capacity of a cell. The rGO@TiO_2_ NFs show very stable cycle lifetimes with high coulombic efficiency (>98%). They show high capacity retention of about 90% after 200 cycles at 1C. Moreover, at 5C, the cycling capacities of the rGO@TiO_2_ NFs increase for about 70 cycles, which indicates a gradual improvement of the electrode kinetics or an increase in the active area. However, the TiO_2_ NFs show a considerably low initial discharge capacity of 131 mAh g^−1^ at 0.2C. Unfortunately, at higher rates, although this 1D nanostructured TiO_2_ may make it possible effectively to transport Na^+^ with regard to the diffusion length, the original insulating property of the TiO_2_ NFs markedly limited the electron transport (47 mAh g^−1^ at a 1C rate and 12 mAh g^−1^ at a 5C rate). For a comparison of the TiO_2_ NFs and rGO@TiO_2_ NFs in light of the power density, the rate performances are shown in Fig. 9d. The rGO@TiO_2_ NFs exhibited high reversible capacities of 217, 182, 164, 146, 119, 87 and 197 mAh g^−1^, while the TiO_2_ NFs showed reversible capacities of 101, 56, 36, 21, 8, 2 and 68 mAh g^−1^ at rates of 0.2C, 0.5C, 1C, 2C, 5C, 10C and recovering rate of 0.2C, respectively. The rGO@TiO_2_ NFs showed 91% of initial capacity at 0.2C when the current density was reversed back to 0.2C, whereas the TiO_2_ NFs exhibited 67% of initial capacity under same condition. The rGO@TiO_2_ NFs clearly show significantly improved rate performances compared to those of the TiO_2_ NFs at all rates. The higher reversible capacities and better cycle performances are evidences that wrapped graphene sheets significantly increase the electrical conduction of TiO_2_ NFs and offer higher electrode conductivity levels with their three-dimensionally interconnecting nanofibers in a complementary manner.

In summary, TiO_2_ NFs with a wrinkled surface and a uniform diameter (200–300 nm) were synthesized via an electrospinning method. To improve the electrical conductivity of the TiO_2_ NFs, rGO sheets were effectively wrapped onto PAH-grafted TiO_2_ NFs. We verified that the sodiation mechanism is clearly based on an insertion process by conducting *ex-situ* XRD and *ex-situ* TEM analyses. The Na anode electrode with rGO@TiO_2_ NFs delivered a high reversible capacity of 217 mAh g^−1^ at 0.2C, excellent cycle performance at 1C (90% capacity retention during 200 cycles) and superior rate capability of 124 mAh g^−1^ at a 5C rate (1.675 A g^−1^). The graphene-wrapping assisted by the surface grafting of the TiO_2_ NFs offers a versatile way to improve the electrical conductivity and electrochemical stability of TiO_2_ NFs for application to Na ion batteries.

## Methods

### Materials

The titanium (iv) isopropoxide (C_12_H_28_O_4_Ti, 98%), polyvinylpyrrolidone (PVP, M_w_ = 1,300,000), dimethylformamide (DMF, anhydrous, 99.8%), GO solution (2 ml mg^−1^) and Poly(allylamine hydrochloride) (PAH, M_w_ = 900,000) were purchased from Sigma-Aldrich. Acetic acid (CH_3_COOH, 99.9% (m/m)) was purchased from Junsei. We used all materials without further purification.

### Synthesis of TiO_2_ nanofibers

In a typical process, TiO_2_ NFs were fabricated via electrospinning. First, 2g of titanium (iv) isopropoxide and 1 g of acetic acid, as a precursor and a dissolving catalyst, respectively, were dissolved in 7 g of DMF. Then, 1.2 g of PVP as a sacrificial template was added to the solution. After stirring the precursor solution at 500 rpm for 12 h, the solution was sequentially loaded into a plastic syringe. Under a voltage of 17 kV and a flow rate of 0.3 mL min^−1^, as-spun Ti precursor/PVP composite NFs were obtained. Here, the feeding rate of the solution was 10 μm/min, and a 25-gauge needle was used in the electrospinning condition. Finally, to decompose the matrix polymers and obtain TiO_2_ NFs, the collected as-spun nanofibers were heat-treated at 500 ^o^C for 1 h to decompose the matrix polymer and crystallize the TiO_2_ NFs.

### PAH functionalization and graphene-wrapping

rGO@TiO_2_ NFs were synthesized by the following three methods. First, 1 g of PAH was dissolved into 25 ml of DI water, and an amount of 0.13 g of TiO_2_ NFs was added to this aqueous solution. This solution was mildly stirred for 1 h to functionalize the surface of the TiO_2_ NFs homogeneously into an amine end group. Then, centrifuging, washing and vacuum drying at 80 ^o^C for 6 h followed. Second, an amount of 0.11 g of prepared PAH-TiO_2_ NFs was dispersed in 10 ml of DI water, and 3200 μL of aqueous GO solution was added to this mixture to synthesize GO-coated TiO_2_ NFs as a hybrid anode material. Third, the GO-wrapped TiO_2_ NFs were dispersed in 10 ml of DI water, and 1.5 g of hydrazine monohydrate was added to this solution to obtain rGO@TiO_2_ NFs by reducing GO to rGO.

### Material characterization

The anatase structure of TiO_2_ was investigated by X-ray diffraction (XRD, D/MAX-RB (12KW) and D/MAX-RC (12 kW), Rigaku). The morphologies of the TiO_2_ NFs and rGO@TiO_2_ NFs were observed by a scanning electron microscope (SEM, Philips). The lattice fringe and selected-area electron diffraction (SAED) patterns were obtained by a transmission electron microscope (TEM, Tecnai F30 S-Twin, FEI). Raman spectroscopy was carried out using a LabRAM HR UV/Vis/NIR PL device by Horiba Jobin Yvon, France. The Fourier-transform infrared spectroscopy (FT-IR) analysis was performed using the attenuated total reflection (ATR) method for the GO solution and the KBr-pellet method for the TiO_2_ NFs and the rGO@TiO_2_ NFs in transmission mode on an IFS66V/S & Hyperion 3000, Bruker Optiks, Germany. Carbon contents were measured by an element analysis (EA, Flash 2000 series, Thermo Scientific).

### Electrochemical measurements

The composition of the slurries was 75 wt% active materials, 15 wt% Super P, and 10 wt% polyvinylidene fluoride (PVDF) binder dissolved in N-methyl-2-pyrrolidinone (NMP). The loading of the active material was about 0.85 mg cm^−2^. Using a doctor blade technique, the slurry was coated onto Cu foils to a thickness of 90 μm. Then, overnight vacuum drying at 80 ^o^C followed. Celgard 2032 coin cells were used to assemble half-cells in an argon-filled glove box. To assemble the half-cells of NIBs, Na foils were used as the counter electrodes. Glass microfiber filters (Whatman) were used as the separator. The electrolyte was 1 M of NaClO_4_ in PC including 5 wt% of fluoroethylene carbonate (FEC). To prepare the half-cells for the LIBs, Li foils and Celgard 2400 were used as counter electrodes and as a separator, respectively. The electrolyte was 1 M LiPF_6_ in EC/DEC (1:1 v/v). Cell tests were carried out with a Maccor 4000 battery tester at a voltage window of 0.01 V ~ 2.5 V. Cyclic voltammetry (CV) was performed on a WBCS3000 device by WonATech at a scan rate of 0.5 mV s^−1^ at 0.01 V ~ 2.5 V.

## Additional Information

**How to cite this article**: Yeo, Y. *et al.* Graphene-Wrapped Anatase TiO_2_ Nanofibers as High-Rate and Long-Cycle-Life Anode Material for Sodium Ion Batteries. *Sci. Rep.* **5**, 13862; doi: 10.1038/srep13862 (2015).

## Supplementary Material

Supplementary Information
