# Supplementary material for: Graphene-Wrapped Anatase TiO2 Nanofibers as High-Rate and Long-Cycle-Life Anode Material for Sodium Ion Batteries
Source: Sci Rep. 2015 Sep 10;5:13862. doi: 10.1038/srep13862 (PMC4564728; doi:10.1038/srep13862)
Supplement: Supplementary Information [file srep13862-s1.doc]

Supplementary Information

Graphene-Wrapped Anatase TiO_2_ Nanofibers as High-Rate and Long-Cycle-Life Anode Material for Sodium Ion Batteries

By Yeolmae Yeo, Ji-Won Jung, Kyusung Park, and Il-Doo Kim*

**[Figure S1]**

**Figure S1**. Charge–discharge profiles of formation cycle for (a) TiO_2_ NFs and (b) rGO@TiO_2_ NFs at a rate of 10 mA g^-1^.

**[Figure S2]**

**Figure S2**. Charge–discharge profiles of formation cycle for (a) TiO_2_ NFs and (b) rGO@TiO_2_ NFs for LIBs at a rate of 10 mA g^-1^. Charge and discharge curves of (c) the TiO_2_ NFs and (d) the rGO@TiO_2_ NFs for LIBs at 1C (335 mA g^-1^) rate.

**[Table S1**]

| **Component Name** | **Element %** |
| --- | --- |
| Nitrogen | 0.1064312 |
| Carbon | 1.849457 |
| Hydrogen | 0.2413903 |
